# The Exo-Polysaccharide Component of Extracellular Matrix is Essential for the Viscoelastic Properties of *Bacillus subtilis* Biofilms

**DOI:** 10.3390/ijms21186755

**Published:** 2020-09-15

**Authors:** Santosh Pandit, Mina Fazilati, Karolina Gaska, Abderahmane Derouiche, Tiina Nypelö, Ivan Mijakovic, Roland Kádár

**Affiliations:** 1 Department of Biology and Biological Engineering, Chalmers University of Technology, Kemivägen 10, 412 96 Göteborg, Sweden; pandit@chalmers.se (S.P.); abdder@chalmers.se (A.D.); 2 Department of Industrial and Materials Science, Chalmers University of Technology, 412 96 Göteborg, Sweden; mina.fazilati@chalmers.se; 3 Department of Chemistry and Chemical Engineering, Chalmers University of Technology, 412 96 Göteborg, Sweden; tiina.nypelo@chalmers.se; 4 Department of Aerospace Engineering, University of Bristol, Bristol BS8 1TR, UK; karolina.gaska@bristol.ac.uk; 5 Wallenberg Wood Science Center, Chalmers, 412 96 Göteborg, Sweden

**Keywords:** biofilms, exopolymeric matrix, interfacial rheology, bicone method

## Abstract

Bacteria are known to form biofilms on various surfaces. Biofilms are multicellular aggregates, held together by an extracellular matrix, which is composed of biological polymers. Three principal components of the biofilm matrix are exopolysaccharides (EPS), proteins, and nucleic acids. The biofilm matrix is essential for biofilms to remain organized under mechanical stress. Thanks to their polymeric nature, biofilms exhibit both elastic and viscous mechanical characteristics; therefore, an accurate mechanical description needs to take into account their viscoelastic nature. Their viscoelastic properties, including during their growth dynamics, are crucial for biofilm survival in many environments, particularly during infection processes. How changes in the composition of the biofilm matrix affect viscoelasticity has not been thoroughly investigated. In this study, we used interfacial rheology to study the contribution of the EPS component of the matrix to viscoelasticity of *Bacillus subtilis* biofilms. Two strategies were used to specifically deplete the EPS component of the biofilm matrix, namely (i) treatment with sub-lethal doses of vitamin C and (ii) seamless inactivation of the *eps* operon responsible for biosynthesis of the EPS. In both cases, the obtained results suggest that the EPS component of the matrix is essential for maintaining the viscoelastic properties of bacterial biofilms during their growth. If the EPS component of the matrix is depleted, the mechanical stability of biofilms is compromised and the biofilms become more susceptible to eradication by mechanical stress.

## 1. Introduction

Bacterial biofilms are communities of microbial cells embedded within a matrix of polymers of their own synthesis [1,2,3]. These polymers constitute a biofilm matrix, which is a mix of exopolysaccharides (EPS), proteins, and extracellular DNA [4,5,6,7]. Growth of bacterial biofilm comprises cell division and accumulation of extracellular matrix, leading to the formation of a complex three-dimensional cell-matrix architecture. Initially, bacterial cells are strongly embedded within the matrix, which provides them with protection against mechanical stresses, and consequently prevents detachment of cells from the community surface [5]. In mature biofilms, cells or clusters of cells (microcolonies) detach from the biofilm and colonize other available surfaces to form new biofilms [6]. Biofilm formation is triggered by quorum sensing [8] and other complex regulatory phenomena that sense nutrient availability [9]. The EPS and protein (TasA, TapA, and BslA) are the major part of the rigid extracellular matrix of *B. subtilis* biofilms. The genes involving EPS production are part of *epsA-O* operon [10,11]. It is well demonstrated that eps-defective mutants developed flat colonies and extremely fragile pellicles [11]. These mutant strains are still able to grow in cell chains and are still embedded with extracellular protein matrix in the biofilms [11]. The proteins TasA and TapA, which provide structural integrity to the biofilm matrix, are produced by the three gene operon *tapA-sipW-tasA* (*tapA* operon) [12]. A mutant of TasA was also shown to produce thin pellicles with less complexity in comparison with the wild type, however, the effect on biofilm formation was not as dramatic as that of the *eps*-defective mutants [10]. Another protein BslA produced during biofilm maturation developed a hydrophobic layer on top of the biofilm, where it served as a water-repellent barrier for the community [13]. In addition, extracellular DNA is reported to interact with EPS for the modulation of the 3D architecture of *B. subtilis* biofilm [14].

The complex biofilm microenvironment offers a degree of protection to bacterial cells, allowing them to survive exposure to different types of environmental stress [15,16]. One type of protection offered by the biofilm matrix is based on restricting penetration of toxic agents into the biofilm, ultimately leading to enhanced survival of bacterial cells during infection [17,18]. This protection has been shown to be effective against biocides, components of the host immune response, and antimicrobial agents [19].

Another type of protection that the biofilm matrix offers is the resistance to mechanical stress [20,21]. In natural environments, biofilms are often confronted with mechanical challenges, such as water pressure affecting biofilms in aquatic environments, flow of body fluids or tissue movement affecting biofilms on indwelling medical devices, and toothbrushing and tongue movement affecting biofilms in the oral cavity [15]. To persist in such environments, biofilms need to remain organized under mechanical stress, exhibiting a degree of elasticity, and they also need to be able to adapt in response to mechanical stress, behaving as a viscous material. In other words, biofilms exhibit viscoelasticity; that is, they combine both viscous and elastic material characteristics when undergoing deformation [22]. In the case of high molecular weight polymers, such as EPS, elastic or energy storage mechanisms are owing to macromolecular conformational changes under stress and the ability of polymer chains to return to a preferential conformation once the stress is removed [23,24]. Entanglements contribute to the elastic material response by acting as physical crosslinks [25]. The viscous or loss mechanisms are owing to the ability of polymer chains to escape their conformational confinement by sliding past neighboring molecules under applied stress [23,25]. Biofilms thus exhibit a complex rheological behavior, with the viscoelastic response determined by the bacterial cells, the extracellular matrix, and interactions between the two [15], as well as confinement, both in terms of triggering biofilm formation [26] as well as influencing chain conformation and EPS entanglement [27].

There is a considerable biodiversity among biofilms formed by different bacterial species, but *B. subtilis* strain NCIB 3610 is widely used as a model organism to study the formation and characteristics of bacterial biofilms [28]. Despite extensive knowledge of biofilm matrix composition [29] of *B. subtilis*, the contribution of individual matrix components to the viscoelastic properties of biofilms is not well understood. Previously, changes in elasticity and surface tension have been monitored during biofilm formation under various environmental conditions (changes in pH, temperature, and nutrient availability) using a custom interfacial rheology setup [30]. It has been concluded that the elastic behavior of biofilms is specific to the type of bacterial strain, availability of nutrient, and other environmental circumstances [30,31]. In the mentioned study, it was stipulated that the actual physical stress needed to disrupt a biofilm could be determined by large amplitude oscillatory shear tests (nonlinear viscoelasticity) [30]. Using this approach, the authors concluded that the presence of surfactin, a surfactant protein encoded by the gene *sfrA-A*, has a positive effect on the formation of *B. subtilis* biofilms [30].

In this study, we used interfacial rheology to elucidate the specific contribution of the EPS component of the biofilm matrix to viscoelastic properties. We used two strategies to deplete the EPS component and compare the result with the wild type biofilm. In the first approach, *B. subtilis* was treated with various concentrations of vitamin C during biofilm formation, as vitamin C has been previously shown to inhibit the formation of *B. subtilis* EPS [32]. In the second approach, we disrupted the promoter region of the *eps* operon, which is required for the biosynthesis of *B. subtilis* [11]. In all cases, the changes in viscoelastic properties of biofilms were monitored in real time for 24 h. The biomass and viability of bacteria were also followed during the experiment. Our results indicate that a reduction of the EPS component of the matrix leads to a selective alteration of growth dynamics and ultimately to a dramatic *B. subtilis* biofilm elasticity. This suggests that, if formation of the EPS component of the matrix is prevented or inhibited, the mechanical stability of biofilms will be significantly reduced.

## 2. Results

### 2.1. Viscoelastic Properties Changes over Time during B. subtilis Biofilm Formation

**Figure 1:**
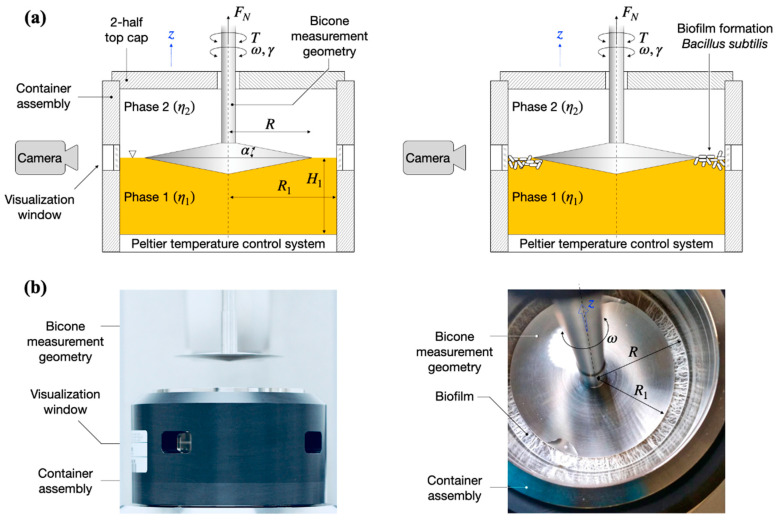
(**a**) Schematic overview of the bicone interfacial rheological system (IRS) and visualization setup. (left) A bicone measurement geometry of radius *R* is placed at the interface between the liquid (lysogeny broth, LB)–gas (air) phases of viscosities $\eta_{1,2}$ in a container assembly of radius *R*_1_. (right) By oscillating the bicone measurement geometry with angular frequency ω and strain amplitude $\gamma_{0}$, the storage and loss moduli, $G^{\prime },G^{\prime \prime }$, of the *B. subtilis* biofilm formed at the LB–air interface in the measurement gap *R*–*R*_1_ are determined based on the torque acting on the bicone, M. A digital camera positioned at a visualization window monitors the biofilm formation. (**b**) Photographs of the interfacial rheological setup: (left) side-view of the setup in the retracted position (out of the container assembly); (right) top view of the setup at the end of a test showing a fully formed biofilm. The 2-half top cap in (**b**) was not included in the photos.

To investigate the changes in viscoelastic properties during the biofilm formation, *B. subtilis* biofilm was grown directly into the interfacial rheology cell container assembly, as shown in the experimental setup overview (Figure 1). The progress in biofilm formation was monitored by taking photographs at 1 h of intervals through a visualization window, Figure 1. We present the development of biofilm viscoelastic properties during growth using the interfacial shear storage (elastic) modulus, $G^{\prime }$, as it is the dominant contribution to the measurement torque ($G^{\prime }\gg G^{\prime \prime }$). The interfacial shear loss (viscous) modulus, $G^{\prime \prime }$, is approximately one order of magnitude lower and generally follows the qualitative behavior of $G^{\prime }$. Prior to the formation of a superficial biofilm layer spanning the interfacial gap ($R_{1}-R$ in Figure 1), the measurement torque is dominated by the bulk lysogeny broth (LB) contribution to the torque (phase 1 in Figure 1) and, therefore, the determined (interfacial) dynamic moduli are below the sensitivity limit of the method. We consider the onset of the biofilm formation from the interfacial rheology data based on the Boussinesque number, defined as follows:

(1) $$Bo=\frac{\eta }{\left(\eta^{\left(1\right)}+\eta^{\left(2\right)}\right)R}$$

where η is the interfacial shear viscosity, $\eta^{\left(j\right)}$ are the upper and lower phase shear viscosities, and R is the bicone radius, see Figure 1. Bo is important for interfacial rheological measurements in defining the measurable limits and the dominant factors determining the interfacial material properties [33]. In the limit of Bo →0, the interfacial flow is dominated by the bulk phases, while in the limit of Bo →∞, the interfacial flow governs the response of the system. For intermediate Bo, the material response contains both bulk, given by phases 1 and 2, and biofilm contributions to the interfacial flow [33] (see also the method description ahead). Defining the lower measurable limits, $Bo_{min}$, of interfacial rheological techniques is not a trivial matter and depends, among others, on the measuring geometry, measurement instrument, and so on, as highlighted in [34,35,36]. In this work, we use $Bo_{min}=1$, as previously used in similar studies in terms of experimental setup and materials [33,37]. Therefore, the onset of biofilm formation according to the interfacial data is quantified as the time after which Bo>1, and the interfacial data reported in the following analysis follow this criterion.

**Figure 2:**
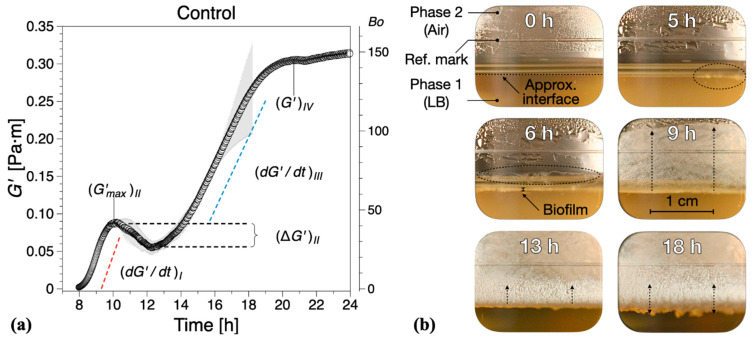
(**a**) Interfacial storage modulus $\left(G^{\prime }\right)$ dynamics corresponding to wild type *B. subtilis* (control) biofilm formation. Data are presented as mean ± standard deviation from two independent biological replicates. The onset of measurable biofilm formation as evidenced by the dynamic moduli (Bo>1) was estimated at 7.8 ± 0.5 h. (**b**) Representative photographs of different stages of biofilm formation through the visualization window. A compilation of the photographs can be found in Supplementary Information Movie 1.

We distinguish four distinct biofilm growth dynamics regions based on the wild type *B. subtilis* (control), Figure 2. The primary growth phase, region *I*, comprised a monotonic increase (see ${\left(dG^{\prime }/dt\right)}_{I}$ in Figure 2a) in the interfacial storage modulus and corresponds to the creation of the superficial biofilm layer spanning the interfacial gap. The increase in the interfacial elastic modulus $G^{\prime }$ in this region is generally consistent with typical adsorption curves [30]. For wild type *B. subtilis* (control)*,* macroscopically visible bacterial aggregation towards the LB medium–air (liquid–air) interface within the visualization window could be observed after 5 ± 1 h; see highlights at 5 h in Figure 2b. After 6 ± 1 h, a complete linage of *B. subtilis* biofilm pellicle spanning the visualization window was apparent, including evidence of side growth on the container wall; see highlights at 6 h in Figure 2b. We note that Bo>1 occurred after 7.8 ± 0.5 h. Discrepancies between the visual observations and interfacial rheological measurements at the onset of biofilm formation can be owing to the limitations in the optical visualization setup and/or measurement sensitivity, as expressed by Bo. Owing to the camera field-of-view limited by the visualization window, the full angular and radial span of the superficial biofilm layer cannot be entirely assessed, that is, whether the biofilm has a full circumferential coverage of the measuring gap. In addition, owing to optical distortions caused by the LB contact angle at the container surface, a precise visual estimation for the formation of a complete lineage of *B. subtilis* biofilm is difficult to make. Incomplete and/or weak superficial biofilms could also lead to weak interfacial contributions to the total torque exerted on the bicone disk. Despite these limitations, we found a reasonable agreement between the optical evidence of biofilm formation and interfacial moduli dynamics. A significant increase in both the superficial layer and on the container walls was observed after 8 ± 1 h, consistent with the steady increase in the interfacial dynamic moduli. After 9 ± 1 h, the entire visualization window was covered by the biofilm grown on the container walls; see 13 and 18 h in Figure 2b.

In region II, a non-monotonic dynamic response was recorded (see ${\left(\Delta G^{\prime }\right)}_{II}={\left(G_{min}^{\prime }-G_{max}^{\prime }\right)}_{II}$ in Figure 2a), whereby the initial $G^{\prime }$ growth (region I) reaches a local maximum ${\left({G^{\prime }}_{max}\right)}_{II}$ after approximately 3 h, decreases to a minimum ${\left({G^{\prime }}_{min}\right)}_{II}$, and then increases again. This local decrease in $G^{\prime }$ has previously been associated to parts of the biofilm being recycled for nutrients as bacteria already embedded within the formed biofilm have limited access to nutrients within the LB medium [30]. The optical visualizations indicate biofilm development to a reduced extent on both the superficial layer as well as in the container walls within the region.

In region *III*, a new monotonic growth in the dynamic moduli was observed—see ${\left(dG^{\prime }/dt\right)}_{III}$ in Figure 2b—as overall biofilm growth becomes once again the dominant dynamic process. The optical visualizations for the region showed significant three-dimensional growth towards both the LB–air interface and container walls; see 13 h in Figure 2b.

Following the monotonic growth in region *III*, a limiting value in interfacial shear storage modulus, $G^{\prime }$, was reached (see ${\left(G^{\prime }\right)}_{IV}$ in Figure 2b), defining region *IV*. This can be considered as a limiting value of the measurable interfacial dynamic moduli for matured biofilms owing to the lowering of the biofilm position due to LB mass loss with respect to the bicone tip. In this region, the behavior in dynamic moduli varied strongly between tests, ranging from plateau-like values to a significant decrease in the dynamic moduli. Such behavior was not take into consideration in calculating the mean and standard deviations of $G^{\prime }$ (same for the following sections). We note that the lowering of the free surface can be attributed to bacteria accumulating at the interface and consuming nutrients from the LB medium, as there are on volatile components in LB. The container was not refilled during tests because of the difficulties in assessing accurately the decrease in surface level and to avoid any disruptions of the surface pellicle. For the control tests, a mature biofilm could be visually observed after 18 ± 1 h growth time, Figure 2b, characterized by an irregular surface texture; see also Figure 1b (right image).

### 2.2. Depletion of the EPS Component of the Matrix Reduces Viscoelasticity of B. subtilis Biofilms

**Figure 3:**
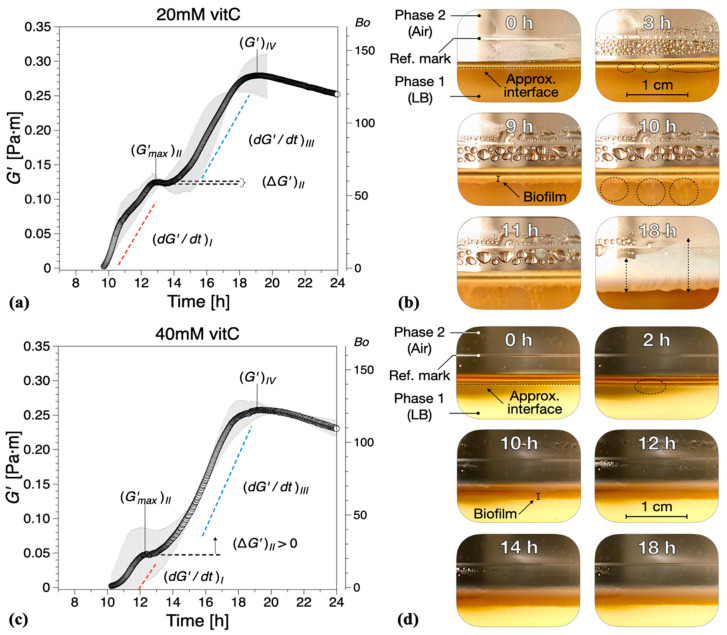

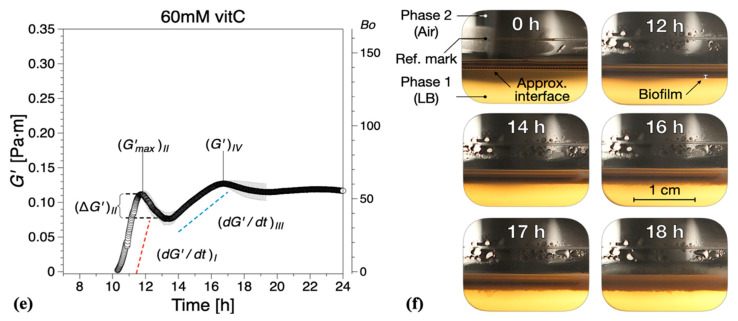
Interfacial storage modulus $\left(G^{\prime }\right)$ and representative photographs as a function of biofilm growth time for *B. subtilis* biofilms grown in the presence of various concentrations of vitamin C (vitC): (**a**,**b**) 20 mM, (**c**,**d**) 40 mM, and (**e**,**f**) 60 mM. The images were chosen based on the time point of visible biofilm formation, surface coverage, and maturation. Data are presented as mean ± standard deviation from two independent biological replicates. The onset of measurable biofilm formation as evidenced by the dynamic moduli (Bo>1) was estimated at (**a**) 9.6 ± 1.3 h, (**c**) 9.5 ± 0.6 h, and (**e**) 10.4 ± 1.5 h. A compilation of the photographs can be found in Supplementary Information Movies 2, 3, and 4.

**Figure 4:**
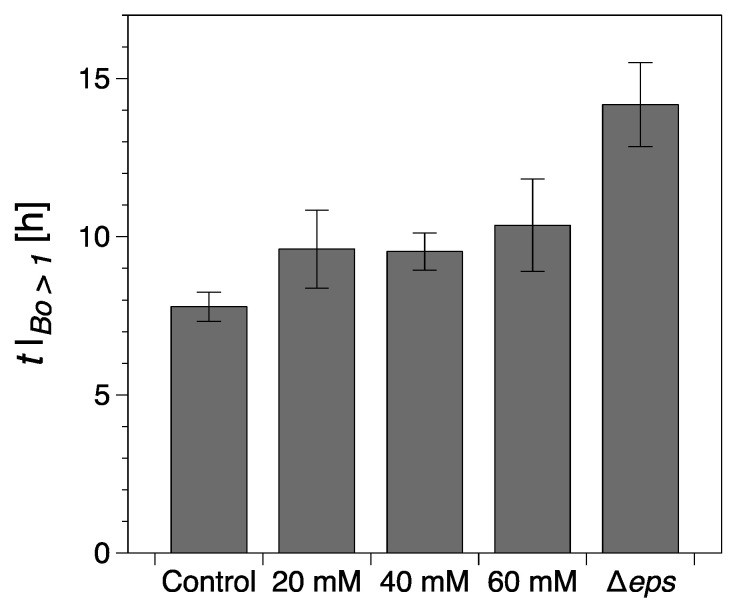
Onset of a continuous surface biofilm as identified through the interfacial rheological measurements (Bo>1). The error bars represent the standard error of the mean. Control refers to the wild type *B. Subtilis* strain (without vitamin C) and Δ*eps* to the eps mutant strain (discussed in the following section).

Vitamin C, in concentrations above 40 mM, is known to drastically reduce EPS production by *B. subtilis* [32]. Hence, we added different concentrations of vitamin C to our experimental setup in order to examine the contribution of EPS to the viscoelastic nature of biofilm growth. The presence of vitamin C in the LB medium significantly affected the dynamics and interfacial properties of biofilms at the LB medium–air interface. It was apparent that, while evidence of surface bacterial aggregates could be spotted early in the optical visualizations, see highlights at 3 h for 20 mM (Figure 3b) and 2 h for 40 mM (Figure 3d), the system does not have the ability to effectively create a continuous surface pellicle. In the presence of increasing concentrations of vitamin C, the initial formation of biofilm on the medium–air interface was delayed. This can be evidenced by both $G^{\prime }$—see Figure 4—and the visual observations.

While the formation of macroscopically observable pellicle occurred at about 6 ± 1 h without vitamin C, this stage was reached after 9 ± 1 h in the presence of 20 mM, after 10 ± 1 h with 40 mM, and after 12 ± 2 h with 60 mM vitamin C added (Figure 3b,d,f).

**Figure 5:**
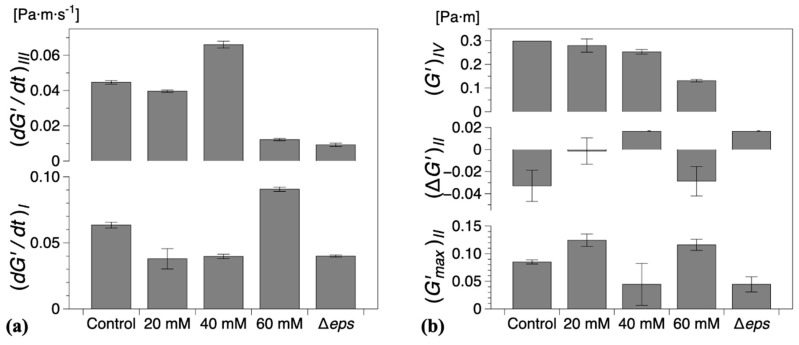
(**a**) Steady growth rates of (mean) interfacial storage modulus ($G^{\prime }$) for regions *I* and *III* and (**b**) characteristic storage modulus for regions *I* and *IV* and ${\left(\Delta G^{\prime }\right)}_{II}={\left(G_{min}^{\prime }-G_{max}^{\prime }\right)}_{II}$ for region *II* for all compositions studied. The error bars represent the standard error of the mean. Control refers to the wild type *B. Subtilis* strain (without vitamin C) and Δ*eps* to the eps mutant strain (discussed in the following section).

The characteristic parameters of growth regions I–IV are compiled in Figure 5. Compared with the control experiments, the presence of vitamin C decreased the growth rates in region *I* ${\left(dG^{\prime }/dt\right)}_{I}$ and/or region *III* ${\left(dG^{\prime }/dt\right)}_{III}$, Figure 5a. Low region *I* growth rates (20 and 40 mM) corresponded to a significant reduction in ${\left(\Delta G^{\prime }\right)}_{II}$, with no decrease in elasticity recorded for 40 mM in region *II* (${\left(\Delta G^{\prime }\right)}_{II}>0)$, Figure 5b. Conversely, for high region *II* growth rates (60 mM), a decrease in elasticity was recorded (${\left(\Delta G^{\prime }\right)}_{II}<0)$, similar to the control experiments, Figure 5b. Most significantly, however, for 60 mM vitamin C, the growth rate in region *III* (${\left(dG^{\prime }/dt\right)}_{III}$) was drastically reduced, Figure 5a, leading to a significant decrease in the interfacial elastic shear modulus ${\left(G^{\prime }\right)}_{IV}$ of the matured biofilms. This corresponds to an overall visual decrease in the thickness and complexity of biofilm of the mature surface biofilm layers. For 40 mM and 60 mM of vitamin C, very thin and fragile biofilms were observed owing to the significant reduction in the synthesis of the EPS matrix.

### 2.3. No EPS Production Disrupts the Dynamics of Viscoelastic Properties during Biofilm Growth

**Figure 6:**
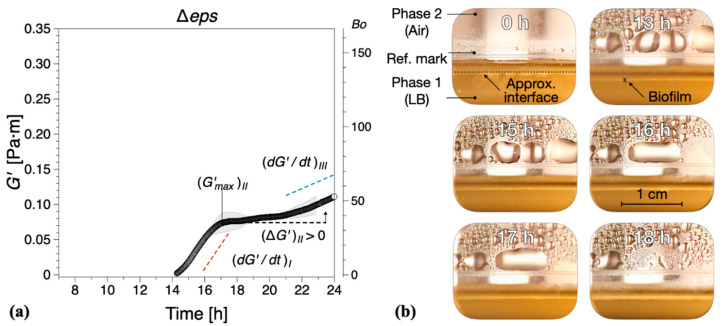
(**a**) The interfacial storage modulus $\left(G^{\prime }\right)$ of biofilm formation by *B. subtilis* Δ*eps* mutant as a function of time. Data are presented as mean ± standard deviation from two independent biological replicates. The onset of measurable biofilm formation as evidenced by the dynamic moduli (Bo>1) was estimated at 14.2 ± 1.3 h. (**b**) Representative photographs of different stages of biofilm formation. A compilation of the photographs can be found in Supplementary Information Movie 5.

Vitamin C leads only to a partial depletion of the EPS component of the *B. subtilis* biofilm matrix, so we asked next what would be the consequence of completely removing EPS. To answer this question, a Δ*eps* mutant of *B. subtilis* NCIB 3610 strain was constructed by disrupting the promoter region of the *eps* operon. Without the expression of *eps* genes, *B. subtilis* is unable to synthesize and export the EPS that normally contribute to the biofilm matrix. Thus, the formation of visible biofilms was significantly altered by the Δ*eps* strain and the dynamic interfacial behavior showed some similarities with the biofilms grown in the presence of vitamin C, Figure 6. The onset of a complete surface biofilm was detected (Bo >1) after 14.2 ± 1.3 h in the dynamic measurements (Figure 4) and 13 ± 1.5 h in the optical visualizations (Figure 6b), significantly retarded compared with the wild type. The growth rates in both regions *I* and *III* (${\left(dG^{\prime }/dt\right)}_{I,III}$) were comparable or lower than the corresponding tests in the presence of vitamin C, with the note that, in contrast to the vitamin C biofilms, both growth rates were significantly reduced when compared with the control results. In addition, no decrease in elasticity was recorded in region *II* ($\Delta G^{\prime }>0$). A limiting interfacial shear elastic modulus ${\left(G^{\prime }\right)}_{IV}$ could not be identified within the experimental time. This is consistent with the resulting Δ*eps* biofilm being very thin, fragile, and smooth, as previously described in the literature [11].

### 2.4. Vitamin C Treatment and Δeps Mutation Reduced the Overall Biomass, but Did Not Significantly Affect Cell Counts Per Volume Unit of Biofilm

**Figure 7:**
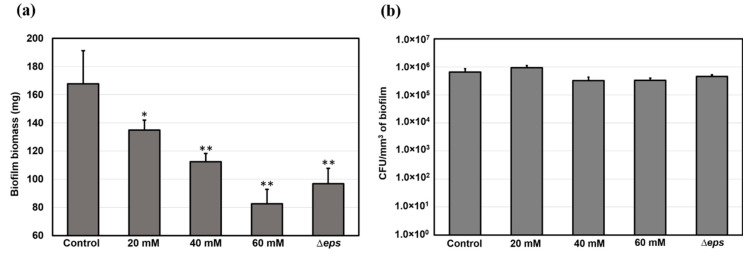
(**a**) Biomass and (**b**) population of bacterial cells in biofilms grown in the presence of vitamin C and biofilm of *eps* mutant. Data represent mean ± standard deviation from three independent biological replicates. * *p* < 0.05, ** *p* < 0.01.

As the thickness, roughness, and overall architecture of the biofilms were altered by vitamin C treatment and the Δ*eps* mutation, we asked whether the depletion of the EPS from the biofilm matrix also affected the number of bacterial cells per volume unit of the biofilm. The total biomass of the films (mass of cells and matrix together) was significantly reduced by vitamin C treatment and Δ*eps* mutation (Figure 7), consistent with our macroscopic observations (Figure 2, Figure 3 and Figure 6). However, the number of bacterial cells, counted as colony forming units (CFUs) per volume unit of biofilm, was found to be comparable in all samples. This control suggests that the measured changes in viscoelasticity are most probably not related to changes in the cell component of the biofilm, but rather directly attributable to the EPS component.

### 2.5. B. subtilis Biofilm Was Fragile without the EPS Component of the Matrix

**Figure 8:**
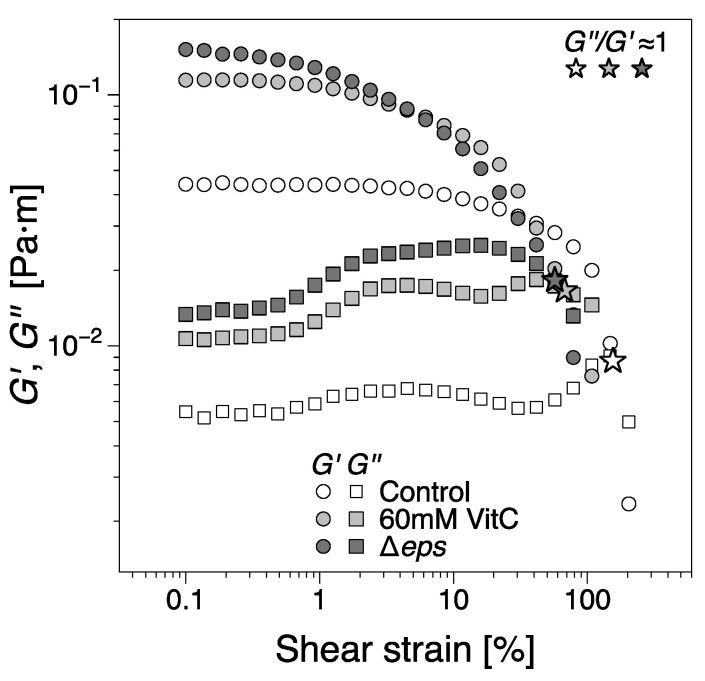
The interfacial shear storage $\left(G^{\prime }\right)$ and loss moduli $\left(G^{\prime \prime }\right)$ of biofilms as a function of shear strain amplitude from oscillatory shear strain sweep tests. The biofilms were tested at the end of the 24 h growth time. The star symbols indicate the approximate location of the crossing between the moduli ($G^{\prime \prime }/G^{\prime }\approx 1)$. The dynamic moduli corresponding to the control biofilms grown for the strain sweep test can be found in Figure S1 (Supplementary Information), as it is an outlier for region IV in the data presented in Figure 2.

To further asses the viscoelastic properties of *B. subtilis* biofilms and their stability, the mature biofilms were subjected to oscillatory shear strain sweep measurements using the same configuration on matured biofilms obtained at the end of the experimental time, Figure 8. While the contact between the geometry and biofilms was firm, there were variations in the distance between the bicone tip and LB level. Furthermore, we note that the data in Figure 2, Figure 3 and Figure 6 are mean values of different experiments and that, as previously noted, the behavior in region *IV* has been particularly prone to large variations in dynamic moduli between tests. Meanwhile, for the least developed biofilms, that is, 60 mM vitamin C and Δ*eps*, there is a reasonable agreement between the dynamic moduli at the end of the 24 h growth time and, at the beginning of the strain sweep tests, there are marked differences for the control (wild type *B. subtilis*) tests. These differences are likely owing to the comparatively significant loss of free surface level resulting in large variations of the dynamic moduli in region *IV*. For reference, the dynamic moduli recorded during the growth of the control biofilm for the strain sweep tests in Figure 8 can be found in Figure S1 (Supplementary Information), showing good agreement with the data in Figure 8. To conclude, overall, the magnitude of the interfacial dynamic moduli cannot be considered for discussion. However, the onset of the nonlinear viscoelastic response (the strain amplitude after which the dynamic moduli are dependent on the strain amplitude) decreased with the addition of vitamin C, and even more for the Δ*eps* strain. This would signify a more ‘brittle’ aggregation of bacteria in the biofilms grown in the presence of vitamin C and Δ*eps* strain, as the increased deformation amplitude easily alters the structure of the biofilm. This is consistent with visual observations, whereby the control biofilm was weakly distorted by the increasing values of strain amplitude. Biofilms grown in the presence of 20 mM vitamin C appeared slightly more distorted, an effect that was more pronounced for 60 mM. The Δ*eps* biofilm was the most unstable. A loss of adhesion between the biofilm and bicone tip/container walls could be observed approximately at the crossover between the dynamic moduli; see the star symbols in Figure 8. This occurred for increasingly lower strain amplitudes for the vitamin C biofilms and even further for the Δ*eps* biofilm. The decrease in strain amplitude for both the onset of nonlinear viscoelastic regime and the loss of adhesion with the walls with increasingly disrupted EPS matrix was expected, as the (high molar mass) EPS matrix confers a certain structural tenacity to biofilms.

## 3. Discussion

As discussed in the introduction, biofilm matrix contains EPS, proteins, and extracellular nucleic acids (mostly DNA) [11,12,37]. The biofilm composition, cells to matrix ratio and ratio of matrix components, changes over time during biofilm development [2,19,38,39,40]. Traditionally, confocal laser microscopy has been used for quantifying the composition and visualizing the 3D architecture and thickness of bacterial biofilms [13,41]. More recently, there has been a focus on understanding the mechanical properties and stability of biofilms by evaluating their rheological properties [42,43,44]. The majority of previous studies focused on assessing the mechanical stability of pre-formed biofilms and pre-formed biofilms that were treated with various disruptive agents [13,45,46,47,48]. Moreover, biofilms of microbial cells with various genes inactivated were evaluated, in order to assess the contribution of those genes to the physical stability of the biofilm [45,46,49]. However, only few studies have examined real-time changes in interfacial rheological properties of bacterial biofilms [30,31]. Interfacial properties are key to biofilm development, interaction with the environment, and ultimately survival [50]. The biofilm surface is known to be crucial for host–bacteria interactions. Stable and impenetrable biofilm surfaces protect the bacteria from the host immune response [50]. This prevents complete eradication of biofilms and promotes antimicrobial resistance in persistent infections. Hence, it is very important to understand the dynamic interfacial rheological properties of these biomatrices during biofilm formation.

Here, we used interfacial rheology via the bicone method in rotational rheometry to study the interfacial shear dynamic moduli of *B. subtilis* NCIB 3610 biofilm surfaces. The dynamics of biofilm formation as characterized by the interfacial elastic shear moduli had previously been shown as a potential ‘fingerprint’ of bacterial type and other environmental factors [30]. We thus identified several growth dynamics regions in the control, wild type *B. subtilis,* biofilms corresponding to the primary growth stage (region *I*), a non-monotonic region where the biofilm experiences a decrease in elasticity signaling; the depletion of nutrients for bacteria embedded in the biofilm (region *II*); the secondary growth stage as overall biofilm growth resumes until biofilm maturation (region *III*); and a final region where the loss in LB mass reduced the level positioning, affecting the measurement magnitude (region *IV*). We hypothesized that the EPS component of the biofilm matrix might have a major contribution to the biofilm viscoelastic properties owing to their polymeric nature. Hence, we depleted the EPS by adding vitamin C [32] and ultimately removed it completely by inactivating the *eps* biosynthetic operon [49]. The disruptions of EPS production had a significant impact on the onset, growth dynamics, and final viscoelastic properties of the biofilms. The formation of a complete biofilm covering the entire interfacial area was delayed by up to 37% (approximately 3 h) for 60 mM vitamin C (Figure 4). This highlights the importance of the EPS matrix as a binding agent of bacteria at the surface. Evidence of biofilm patches was spotted earlier in the growth time, but the system was unable to form a complete pellicle quickly. Vitamin C has shown the ability to selectively disrupt biofilm dynamics in two or more growth regions (Figure 5). The alterations in biofilm formation and growth dynamics ultimately affected the final viscoelastic properties of matured films, as evidenced by their interfacial shear elastic modulus in region *IV* (${\left(G^{\prime }\right)}_{IV}$; Figure 5) and their linear–nonlinear transition in strain sweep tests (Figure 8). As noted in the previous sections, with the consumption of nutrients and accumulation of bacteria at the interface, the free surface is progressively lowered compared with the bicone tip. As the bacteria loses the ability to produce EPS and the biofilm growth is disrupted, the loss of free surface level is expected to become progressively diminished as the EPS secretion is lowered. This can be confirmed by comparing the evolution of the approximate free surface in the optical visualizations between Figure 2b, Figure 3b,d,e and Figure 6b. Therefore, the changes in growth dynamics are likely to be even more pronounced than the values reported in Figure 5. The fact that the development of the interfacial shear elastic modulus did not affect all films in the same growth stages could signify that the EPS molecular properties, for example, molecular weight, entanglement, and chain conformation, play an important role in biofilm formation mechanisms and need to be further explored. The role and influence of the EPS matrix were confirmed by the results for the *eps* mutant strain showing a similar behavior to the higher vitamin C concentration investigated. The outcomes were specifically owing to the depletion of EPS from the biofilm matrix, and not to a loss of cell density per unit of biofilm volume (Figure 7). This result is consistent with a previous study demonstrating loss of elastic behavior of pre-formed *eps* mutant biofilms [45].

Overall, the present study thus further confirms the viability of vitamin C to inhibit growth and degrade the mechanical properties of bacterial biofilms, and we demonstrate that the EPS component of the matrix is crucial for the viscoelastic properties of the biofilm surface. There appear to be structure–property relationships during biofilm formation that could be significant from a mechanical point of view, an important aspect for maintaining mature biofilms films with detrimental mechanical stress. Therefore, our study strengthens already available evidence that the EPS synthesis should be considered as one of the major targets to eradicate persistent biofilm-based bacterial infections [51,52].

## 4. Materials and Methods

### 4.1. Bacterial Strains and Culture Medium

*B. subtilis* NCIB 3610 (Bacillus Genetic Stock Center; Columbus, USA) was used in this study to monitor mechanical properties and stability of biofilms. Cell cultivation was performed in liquid LB medium (10 g of tryptone, 5 g of yeast extract, 5 g of NaCl per liter of broth) or solid LB medium supplemented with 1.5% of agar. The *eps* mutant was constructed using pMUTIN 2 vector to disrupt the promoter region of the *eps* operon and the first two genes, *epsA* and *epsB*. As a result, the entire *eps* operon is inactivated and the insertion of the vector into the chromosome confers erythromycin resistance. The mutant strain was grown in the LB medium containing erythromycin (1 µg/mL). Sodium ascorbate (vitamin C) was purchased from Sigma–Aldrich (St. Louis, MI, USA)

### 4.2. Biofilm Formation and Interfacial Rheology

Interfacial rheology measurements were carried out using Anton Paar MCR702 TwinDrive rheometer (Graz, Austria) in single drive mode equipped with an interfacial rheology system (IRS). *B. subtilis* biofilms were formed in an IRS container using liquid LB media with or without the presence of vitamin C (final volume was 104 mL). Then, 2–5 × 10^6^ CFU/mL of overnight grown bacterial culture was used to inoculate fresh LB medium in the rheometer container. The control biofilms of *B. subtilis* were grown in LB media without vitamin C. The temperature was set to 37 °C to favor the bacterial growth and a bicone geometry (bicone diameter: 68.214 mm, cup diameter: 80.000 mm, cone angle: 5.012°, penetration depth: 2.201 mm) was used. We note that the temperature is set and measured on the bottom plate of the setup; see the Peltier temperature control system in Figure 1a. Therefore, in the absence of a temperature probe in the container, there may be temperature differences between the bottom plate and the interface. To ensure a uniform temperature inside the container, the container assembly includes a thermal insulating sleeve (dark cover in Figure 1b) and a 2-half top cap to seal off the container environment. The tip of the bicone was set within the media–air interface as shown in Figure 1 using a custom procedure for the precise determination for the free surface using the normal force ($F_{N}$ in Figure 1) detected by the instrument. A Canon 60D DSLR camera (Tokyo, Japan) equipped with a Cannon L-Series 100 mm macro lens was positioned at one of the visualization windows (Figure 1). Pictures were taken at 1 h intervals using a remote data acquisition setup. The biofilm formation was monitored through the real-time changes in viscoelastic properties (described below) and visual observations for 24 h.

In strain-controlled steady shear rotational (bulk) rheometry [53,54], a shear rate, $\dot{\gamma }$, is imposed based on analytical solutions of the equations of motion, the angular velocity Ω, and the dimensions of the measurement geometry. The measured variable is the torque exerted on the measurement geometry, M, and is converted into stress, which, together with the imposed shear rate, forms the basis for the calculation of rheological properties. A similar analysis is performed in dynamic tests, where the imposed variables are time-dependent and are characterized by an angular frequency, ω, and strain, γ. Owing to the complexity of the governing equations, in the particular case of the interfacial rheology bicone system, the data acquisition and the determination of the interfacial rheological properties are done separately [33]. Here, we present a summary of the governing equations as a procedure to obtain the measured quantities elaborated in the present work. For the measurement geometry notations, see Figure 1. A more detailed description can be found elsewhere [33,55,56,57]. The interfacial velocity distribution, $v_{\theta }^{\left(\sigma \right)}$, is determined based on the following integral equation of the first kind in dimensionless form [33,58]:

(2) $$\bar{r}{\bar{v}}_{\theta }^{\left(\sigma \right)}\left(\bar{r}\right)={\bar{R}}^{2}\frac{1-{\bar{r}}^{2}}{1-{\bar{R}}^{2}}+\frac{1}{Bo}\int_{0}^{1}K\left(x,\bar{r}\right){\bar{v}}_{\theta }^{\left(\sigma \right)}\left(x\right)dx,$$

where ${\bar{v}}_{\theta }^{\left(\sigma \right)}=v_{\theta }^{\left(\sigma \right)}\Omega /R-\bar{r}$ is the dimensionless interfacial velocity distribution (azimuthal component), $\bar{r}=r/R_{1}$ is the dimensionless radial coordinate, $\bar{R}=R/R_{1}$ is the dimensionless bicone radius, Bo is the Boussinesq number (Equation (1)), and the Kernel function:

(3) $$K\left(x,\bar{r}\right)=\frac{1-{\bar{r}}^{2}}{1-{\bar{R}}_{1}^{2}}G\left(x,\bar{R}\right)-G\left(x,\bar{r}\right)$$

with

(4) $$G\left(x,y\right)=2xy\sum_{i=1}^{\infty }\frac{C_{i}J_{1}\left(\xi_{i}x\right)J_{1}\left(\xi_{i}y\right)}{\xi_{i}J_{0}\left(\xi_{i}\right)}$$

where $J_{0,1}$ are the Bessel functions of the first kind of orders zero and one, $\xi_{i}$ are their zeros, and the coefficients $C_{i}$ are defined as

(5) $$C_{i}=\frac{Y}{1+Y}\coth\left(\xi_{i}{\bar{H}}_{1}\right)+\frac{1}{1+Y}\coth\left(\xi_{i}\left[{\bar{H}}_{2}-{\bar{H}}_{1}\right]\right)$$

where $Y=\eta^{\left(1\right)}/\eta^{\left(2\right)}$ is the bulk viscosity ratio and ${\bar{H}}_{1}=H_{1}/R_{1}$, ${\bar{H}}_{2}=H_{2}/R_{1}$ are the dimensionless depths of the two phases. We note that Bo in Equation (2) contains the interfacial viscosity and the viscosities of phases 1 and 2. In the limit cases, (i) Bo ≪1, the interfacial flow is dominated by the two bulk phases; and (ii) Bo ≫1, the interfacial flow is decoupled from the bulk phases 1 and 2 and they can be neglected. Intermediate Bo numbers pertain to the contribution of both interface and bulk phases to the interfacial flow.

The corresponding dimensionless total torque can be then computed as [34]

(6) $$\bar{M}=\frac{2Bo}{1-{\bar{R}}^{2}}\left[{\bar{R}}^{2}+\frac{1}{Bo}\int_{0}^{1}G\left(x,\bar{R}\right){\bar{v}}_{\theta }^{\left(\sigma \right)}\left(x\right)dx\right]$$

The (imposed) angular velocity and torque are converted into interfacial shear rate, $\dot{\gamma }$; interfacial shear stress, σ; and the resulting interfacial shear viscosity, η, based on solving Equations (1)–(5). For dynamic oscillatory measurements, the angular velocity is varied sinusoidally, resulting in a sinusoidal torque output shifted by a phase angle. Consequently, the interfacial shear strain and stress are in complex notation $\gamma^{*}=\gamma_{0}e^{i\omega t}$ and $\sigma^{*}=\sigma_{0}e^{\left(i\omega t+\delta \right)}$, where $i=\sqrt{-1}$, $\gamma_{0},\sigma_{0}$ are the interfacial shear strain and shear stress amplitudes, ω is the angular frequency, and δ is the phase shift angle. The complex interfacial shear modulus can thus be defined as

(7) $$G^{*}\equiv \frac{\sigma *}{\gamma *}=\frac{\sigma_{0}}{\gamma_{0}}e^{i\delta }=G^{\prime }+iG^{\prime \prime }$$

where $G^{\prime }\equiv \sigma_{0}/\gamma_{0}\cos\delta$ and $G^{\prime \prime }\equiv \sigma_{0}/\gamma_{0}\sin\delta$ define the interfacial shear storage and loss moduli used to characterize the biofilms in this publication.

The interfacial rheological data in Equations (1)–(7) were computed using the analysis routine implemented into the Anton Paar RheoCompass software with the following measurement and analysis input parameters: strain amplitude $\gamma_{0}=0.1\%$; angular frequency ω/2π=1 Hz ([30]); phase (1) (air) complex viscosity $\left|\eta^{\left(1\right)}\right|=1.8\cdot 10^{-5}\;\mathrm{Pa}\cdot s$; and phase (2) (LB) complex viscosity $\left|\eta^{\left(1\right)}\right|=0.062\;\mathrm{Pa}\cdot s$ (determined from trial tests). The heights of the respective phases were determined on the basis of the bicone tip position after completing the automated free surface detection using the normal force sensor of the rheometer prior to each test.

### 4.3. Biofilm Disruption Analysis with External Stress

To further analyze the shear dynamic behavior of bacterial biofilms, *B. subtilis* biofilms were grown in LB media, in the presence and absence of vitamin C and for the Δ*eps* strain. The biofilms were grown in the same conditions as described in Section 4.2 and the strain sweep tests were performed on the matured biofilms at the end of the 24 h growth time. The imposed angular frequency, ω, was 2 rad/s and the shear strain amplitude, $\gamma_{0}$, varied between 0.1 and 200%. The disruption in biofilms was examined by monitoring the changes in elastic and loss modulus.

### 4.4. Viability of Bacteria and Biofilms’ Biomass

Viability of bacteria in biofilms was evaluated by the CFU counting method. Briefly, *B. subtilis* biofilms were collected in 10 mL of 0.89% of sterile NaCl from the rheometer container after the 24 h of interfacial rheology measurement and sonicated at 10 W for 30 s to homogenize the biofilm. Then, 100 µL of homogenized suspensions was serially diluted and plated on LB agar plates to count the colonies. The counted numbers of colonies were normalized with the volume of respective biofilms. Remaining homogenized suspension was washed three times (5000 g for 20 min) with sterile water, lyophilized, and weighed to determine the total biomass of biofilms.

## Supplementary Materials

The supplementary materials are available online at https://www.mdpi.com/1422-0067/21/18/6755/s1.

## Abbreviations

EPS	Extracellular polymeric substances
CFU	Colony forming unit
eDNA	Extracellular deoxyribonucleic acid
LB	Lysogeny broth
