# Supplementary material for: The Exo-Polysaccharide Component of Extracellular Matrix is Essential for the Viscoelastic Properties of Bacillus subtilis Biofilms
Source: Int J Mol Sci. 2020 Sep 15;21(18):6755. doi: 10.3390/ijms21186755 (PMC7555405; doi:10.3390/ijms21186755)
Supplement: Supplementary file 1 [file ijms-21-06755-s001.pdf]

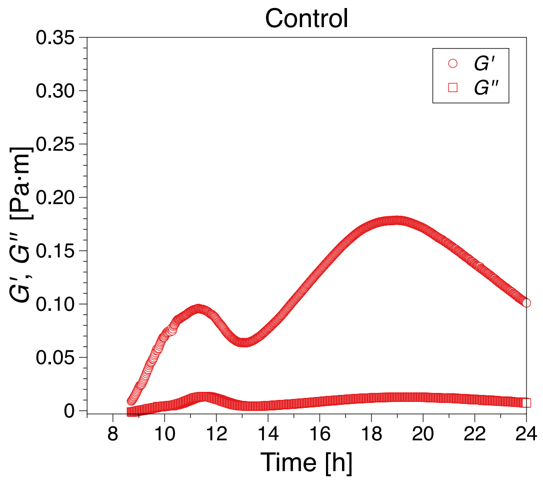

Figure S1. Dynamic moduli, G' *and* G'', of the Control biofilm grown for the strain sweep test in Figure 8. Note the pronounced decrease in dynamic moduli in region IV, decrease that was not accounted for in the standard deviation calculations in Figure 2.
